# Internal herniation of the abdominal wall

**DOI:** 10.1002/ccr3.2008

**Published:** 2019-01-29

**Authors:** Arthur Bloemen, Marlies J. Keijzers, Joop L. M. Konsten, Frits Aarts, F. Jeroen Vogelaar

**Affiliations:** ^1^ Department of Surgery VieCuri Medical Centre Venlo The Netherlands

**Keywords:** abdominal wall defect, anatomical variation, arcuate line, hernia

## Abstract

A symptomatic arcuate line hernia should be considered in patients with acute lower abdominal complaints. This rare internal herniation is caused by a sharp ending of the posterior aponeurotic sheath of the rectus muscle, rather than the more common gradual delineation, and can cause strangulation or incarceration of abdominal contents.

## 1 QUESTION

**Figure 1:**
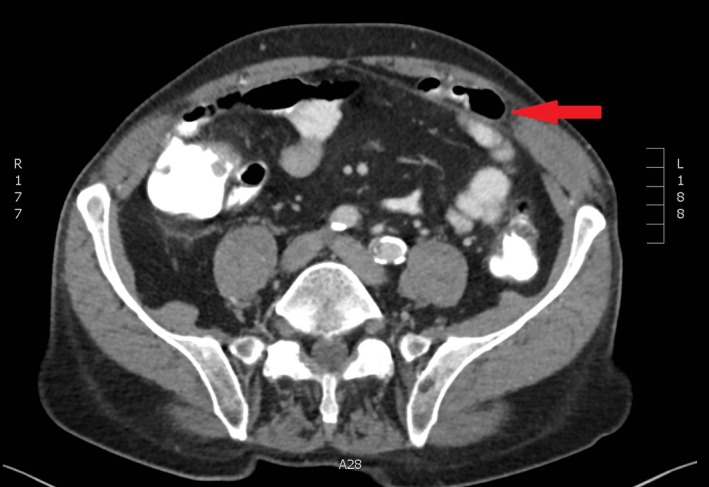
Transversal image of CT scan showing incarcerated herniation in the lower left quadrant of the abdomen (arrow)

**Figure 2:**
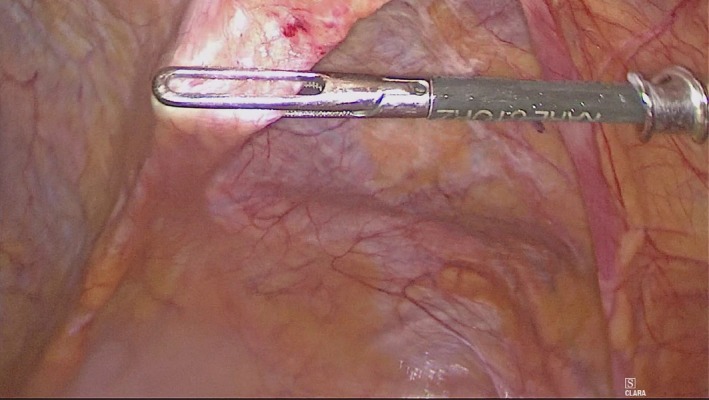
Intra‐operative findings during laparoscopy (Cranial side of patient on left side of image; left flank of patient on lower side of image)

A 79‐year‐old male undergoes exploratory laparoscopy because of an obstructive ileus with suspected incarcerated ventral hernia on CT scan of the abdomen (Figure 1). Intra‐operative findings are shown in Figure 2.

Which type of anatomical variation was found?

## 2 ANSWER

An arcuate line hernia was found (Figure 2). Arcuate line hernias are rare internal herniations caused by a sharp end of the posterior aponeurotic sheath of the rectus muscle, rather than the more common gradual delineation of the sheath. A peritoneal fold with duplication behind this aponeurotic rim may form, creating an internal herniation.

The prevalence of arcuate line hernias is low. In a retrospective review of computed tomography in 315 unselected patients, arcuate line hernia was found in 2.2% of the patients.^1^

Treatment consists of reduction of the hernia with mesh placement over the defect. Laparoscopic approach is preferable because this allows inspection of hernia contents and concomitant hernias, and allows for intra‐ or preperitoneal mesh placement to cover the defect.^2^

Although rare, symptomatic arcuate line hernias should be considered in patients presenting with complaints of the lower abdomen.

## CONFLICT OF INTEREST

None declared.

## AUTHOR CONTRIBUTION

AB: contributed to writing and editing of the manuscript and literature search. MK: involved in writing and editing of the manuscript and editing images and figures. JK: contributed to proofreading of the manuscript and literature search. FA: involved in proofreading of the manuscript and literature search. FJV: contributed to proofreading of the manuscript and editing images and figures.
